# Transdisciplinary experiential learning in biomedical engineering education for healthcare systems improvement

**DOI:** 10.1186/s12909-023-04171-x

**Published:** 2023-04-03

**Authors:** Luis Montesinos, David Ernesto Salinas-Navarro, Alejandro Santos-Diaz

**Affiliations:** 1 Institute of Advanced Materials for Sustainable Manufacturing, Tecnologico de Monterrey, Mexico City, 14380 Mexico; 2 School of Engineering and Sciences, Tecnologico de Monterrey, Mexico City, 14380 Mexico; 3 Aston Business School, Aston University, Birmingham, B4 7ET UK; 4 School of Medicine and Health Sciences, Tecnologico de Monterrey, Monterrey, 64710 Mexico

**Keywords:** Biomedical engineering, Engineering education, Transdisciplinary learning, Experiential learning, Healthcare management engineering, Lean healthcare, Higher education, Educational innovation

## Abstract

**Background:**

The growing demand for more efficient, timely, and safer health services, together with insufficient resources, put unprecedented pressure on health systems worldwide. This challenge has motivated the application of principles and tools of operations management and lean systems to healthcare processes to maximize value while reducing waste. Consequently, there is an increasing need for professionals with the appropriate clinical experience and skills in systems and process engineering. Given their multidisciplinary education and training, biomedical engineering professionals are likely among the most suitable to assume this role. In this context, biomedical engineering education must prepare students for a transdisciplinary professional role by including concepts, methods, and tools that commonly belong to industrial engineering. This work aims to create relevant learning experiences for biomedical engineering education to expand transdisciplinary knowledge and skills in students to improve and optimize hospital and healthcare care processes.

**Methods:**

Healthcare processes were translated into specific learning experiences using the Analysis, Design, Development, Implementation, and Evaluation (ADDIE) model. This model allowed us to systematically identify the context where learning experiences were expected to occur, the new concepts and skills to be developed through these experiences, the stages of the student’s learning journey, the resources required to implement the learning experiences, and the assessment and evaluation methods. The learning journey was structured around Kolb’s experiential learning cycle, which considers four stages: concrete experience, reflective observation, abstract conceptualization, and active experimentation. Data on the student’s learning and experience were collected through formative and summative assessments and a student opinion survey.

**Results:**

The proposed learning experiences were implemented in a 16-week elective course on hospital management for last-year biomedical engineering undergraduate students. Students engaged in analyzing and redesigning healthcare operations for improvement and optimization. Namely, students observed a relevant healthcare process, identified a problem, and defined an improvement and deployment plan. These activities were carried out using tools drawn from industrial engineering, which expanded their traditional professional role. The fieldwork occurred in two large hospitals and a university medical service in Mexico. A transdisciplinary teaching team designed and implemented these learning experiences.

**Conclusions:**

This teaching-learning experience benefited students and faculty concerning public participation, transdisciplinarity, and situated learning. However, the time devoted to the proposed learning experience represented a challenge.

## Background

The growing demand for more efficient, timely, and safer healthcare services, together with insufficient resources, put unprecedented pressure on healthcare systems around the world [1]. This challenge has created new opportunities to improve and optimize healthcare services from a transdisciplinary perspective. In other words, some healthcare organizations and services have adopted the principles and practices of lean manufacturing to maximize value and reduce waste, spawning the concept of lean healthcare [2]. Accordingly, countries such as the United States of America (USA) [3], Canada [4], and the United Kingdom (UK) [5] have designed and implemented local, regional, and national programs of this nature with satisfactory results. However, the design and implementation of this type of program require professionals capable of crossing the traditional boundaries of their discipline to have a broad knowledge of healthcare institutions, their concerns and limitations, and the necessary methods and tools to bring about effective and efficient solutions [6].

Biomedical engineering professionals are among the most suitable to assume this role, given their multidisciplinary education and training [6]. Biomedical engineering ‘integrates physical, mathematical and life sciences with engineering principles for the study of biology, medicine, and health systems and for the application of technology to improve health and quality of life’ [7]. Traditional education initially prepares biomedical engineering students in the fundamentals of anatomy, physiology, and classical engineering (e.g., mechanical, electrical, and computer engineering). Later, it allows them to specialize in areas such as bioinstrumentation and biosignal processing, biomechanics and biomaterials, rehabilitation engineering, medical imaging and radiology, medical and health informatics, and clinical engineering, among others [8]. Despite biomedical engineering education and training around the world sharing a common body of knowledge, some differences in its evolution rate across geographical regions have been identified by the World Health Organization (WHO), with the developed countries leading the way to a broader and more responsive to industry and research latest developments curriculum [8].

From a practice perspective, biomedical engineering professionals are employed in the health technology and healthcare industries, hospitals and other healthcare organizations (e.g., ambulatory surgical centers, dialysis services, and imaging and radiology facilities), academia, government institutions, and national regulatory agencies (e.g., the Food and Drug Administration in the USA and the European Medicines Agency in the European Union) [9]. When working in healthcare institutions, their role as clinical engineers includes selecting, installing, and managing medical equipment and technology; planning clinical areas for healthcare delivery; supporting other healthcare professionals in the appropriate use of diagnostic, treatment, and rehabilitation technologies; and developing specialized or customized instruments or devices for research or treatment [9]. Consequently, the International Labour Organization included professionals in “biomedical engineering” as part of the health workforce along with those occupations classified in sub-major Group 22: Health professionals in the International Standard Classification of Occupations published in 2008 (ISCO-08) [10].

Given adequate education and training, biomedical engineering professionals could positively impact at least three health quality domains: safety, timeliness, and efficiency [1]. In 2017, a group of field experts commissioned by the WHO stressed that biomedical engineers must play a critical role in the evolution of healthcare systems. This group of experts also proposed a list of topics to advance biomedical engineering education and professional development in this direction, including clinical process modeling and reengineering, Six Sigma, lean manufacturing, and root cause analysis [6]. Therefore, biomedical engineering education must prepare students to perform a transdisciplinary professional role by including concepts, methods, and tools that commonly belong to industrial engineering, involving problem-solving and decision-making related to the improvement and optimization of processes and operations required for the production of products and services [11]. In other words, a new professional role for biomedical engineers would involve the integration of professional activities related to medical equipment and technology with those associated with the efficient and timely flow of patients and information, the provision of safe and high-quality services, and the reduction or elimination of inefficiencies and waste in operations [12, 13]. By doing this, biomedical engineers can provide comprehensive socio-technical engineering solutions within healthcare organizations of the 21st century concerning biomedical technologies and ground these into the production and delivery of healthcare services [14].

On the other hand, several mechanisms have been identified to provide biomedical engineering students with hands-on experiences throughout the curriculum, promoting their readiness to pursue careers in the industry while increasing their learning and participation [15]. These mechanisms include computer simulation, laboratory experiments, design courses, guest speakers, industry-sponsored design projects, field trips to hospitals and medical device companies, and internships. Furthermore, some instructional methods have been proposed to improve biomedical engineering education, including problem-based, project-based, challenge-based, and experiential learning, particularly in North American higher education institutions [16–22]. These methods engage students in collaboratively developing solutions to real-world problems based on crucial concepts in the discipline, thus fostering disciplinary knowledge and creative thinking skills. Furthermore, these methods have been found to increase student motivation and awareness of the connections between their in-class experiences and future work [23].

This work addresses the above opportunity by creating relevant learning experiences for biomedical engineering education to develop transdisciplinary knowledge and skills that support students’ future professional careers in complex healthcare systems, emphasizing improving and optimizing hospital and healthcare processes. To advance in this direction, it is also necessary to involve teaching and learning approaches to enhance learning effectiveness through relevant and engaging educational experiences [24–26]. Accordingly, this paper describes the design and implementation of experiential learning activities in healthcare operations management and lean systems to prepare biomedical engineering students to diagnose, evaluate and design process improvements based on industrial engineering methods and tools, emphasizing systems modeling, continuous improvement, lean systems, and operations management. Ultimately, this paper aims to disseminate the authors’ experience in instructional design to advance biomedical engineering education with new concepts and tools through active learning experiences. However, it does not intend to assess its impact on learning effectiveness or make inferences about the results obtained.

This work fosters transdisciplinary learning, a form of education that transcends traditional disciplinary boundaries and integrates multiple disciplines and perspectives into the learning process. This approach encourages students to draw on knowledge and skills from different fields to solve complex problems and often involves project-based learning or other forms of hands-on, experiential learning [27]. Transdisciplinary learning is particularly relevant in today’s healthcare systems, where many of the most pressing public health issues require solutions that draw on expertise from multiple disciplines [28]. Accordingly, this work draws on experiential learning theory to design and implement learning experiences to prepare biomedical engineering students to improve and optimize hospital and healthcare processes. Together, transdisciplinary and experiential learning can be a powerful combination. They provide students with opportunities to apply their learning in real-world contexts and engage with complex problems that require a multidisciplinary approach. This approach can help bridge the gap between theory and practice and prepare students for the challenges they will face in their careers and lives more broadly. Therefore, we describe below the ideas behind experiential learning for biomedical engineering as follows: (i) presenting the main ideas of experiential learning to support the design of biomedical engineering transdisciplinary learning experiences, and (ii) a framework to guide the design of purposeful biomedical engineering transdisciplinary learning experiences about relevant real-world situations related to the improvement and optimization of healthcare and hospital processes.

### Experiential learning for BME education

The traditional view of higher education consists of faculty lecturing students who passively listen and take notes [29–31]. Consequently, student learning and progression in their education are measured through exams, reports, and the hours spent in classrooms [29]. However, this approach is a highly ineffective way of learning, as it promotes a broadcasting type of knowledge delivery in which teaching, not learning, is what matters. Hence, lecturers play a central role while students take a peripheral part in which they passively listen, write, and memorize.

Experiential learning is claimed to overcome this limitation by improving the motivation to learn through the active participation of students in their learning activities to construct learning within their conditions and reality [32]. Therefore, experiential learning requires a different understanding of learning experiences and an action framework to conceptualize, organize, and implement meaningful activities in real-life environments and reflective practice. Moreover, experiential learning has become very important for other active approaches to developing skills in students and their learning outcomes, such as service learning, challenge-based learning, and competency-based education [33].

**Fig. 1:**
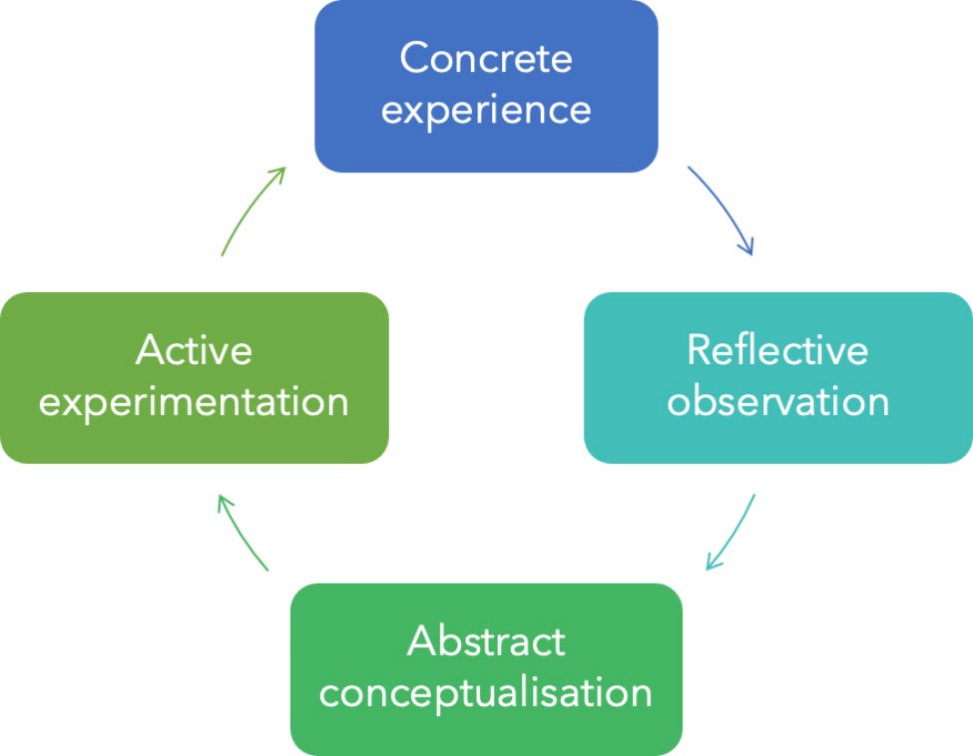
Kolb’s experiential learning cycle. Adapted from [36]

Experiential learning is a constructivist theory highlighting how learners construct knowledge in their learning activities to achieve their intended learning outcomes [34]. From this point of view, teaching is not about broadcasting information but rather engaging students in building their knowledge [35, 36]. Accordingly, the experiential learning approach involves an integrated four-stage loop process that begins with a here-and-now experience, followed by observation and collection of data on the experience, data analysis and formulation of conclusions, and modification of behaviors and selection of new experiences [32, 34–36]. This iterative learning cycle concerns *concrete experience* or perceiving in a situation, *reflective observation* or assessment, *abstract conceptualization* or mapping/design, and *active experimentation* or implementation of situated actions (Fig. 1). According to Kolb, this type of learning occurs as part of a naturally continuous meaning-making process through personal and environmental experiences [36].

Experiential learning highlights taking individual students to situations where they can build their abilities, such as problem-solving, decision-making, and critical thinking [37–40]. This perspective is essential in engineering education to address immersive, hands-on interventions and even simulations that involve the development of technological solutions (see, for example, [41–44]).

Consequently, experiential learning involves defining and organizing learning activities following the recursive cycle [36]. By conducting experiential learning activities, students can increase their interest, motivation, and engagement through different learning choices and paths and be autonomous [32, 45, 46]. Therefore, experiential learning contributes to learners making a stronger connection between learning participation, practices, and the reality [44, 47, 48].

Furthermore, since learning takes place within experiences, these can be considered a wide variety of purposeful immersions or activities in situations in different contexts and settings that transform the perceptions of the learner, facilitate conceptual understanding, produce emotional qualities, and nurture the acquisition of knowledge, skills, and attitudes aligned with a set of intended learning outcomes [49]. Rasiah et al. indicate that learning experiences are ideally challenging, interesting, rich, engaging, meaningful, and appropriate for the learner’s needs [50]. Finally, since learning experiences are cumulative, previous learning experiences determine or condition future learning engagements.

However, designing an effective learning experience implies meeting specific requirements to fulfill its aim [36]: (i) the learner must be actively involved in a relevant experience and receive guidance on *what to observe* and *how to observe* according to a reference knowledge framework (for instance, the learning objectives dictated by their courses or modules); (ii) the learner must be induced to reflect upon the experience, for instance, through conversations with colleagues, tutors or herself; (iii) the learner must be guided on how to conceptualize the experience, for example, in terms of models, maps, or frameworks; and (iv) the learner must be challenged to propose alternatives or solutions to a given problem, issue or concern to foster their decision-making and problem-solving skills.

These previous ideas have powerful implications for designing learning experiences covering the intended learning objectives and expected outcomes in a module or a course. Learning experiences should preferably involve relevant real-world situations linked to the students’ reality or accessible validated experiences, support learning by doing, give students an active role and responsibility, and provide continuous feedback to improve student performance.

Other educational approaches complement experiential learning, given their active and hands-on focus. For example, this is the case for competency-based education, collaborative learning, and challenge-based learning, setting specific requirements to design learning experiences [51]. Consequently, experiential learning can be regarded as a general learning strategy for active learning to learn from experiences. In contrast, competency-based education defines the competencies or skills to develop in students, and challenge-based learning considers the active participation of students in real-world, relevant, and externally linked situations involving a challenging problem and developing alternative solutions. Furthermore, problem-based learning allows students to focus on problem-solving with hands-on activities. Finally, collaborative learning can help take individual learning to produce collective learning from teamwork and meaningful student interactions [52]. Therefore, this work proposes the design of relevant active experiences, in alignment with other educational approaches, by integrating the four-stage experiential cycle to achieve learning objectives and develop particular skills and learning outcomes for the education of BME.

### A framework for mapping healthcare processes for biomedical engineering education

Since biomedical engineering education requires going beyond traditional biomedical engineering activities to expand skills acquisition and professional development, this work elaborates on the definition of relevant learning experiences within healthcare and hospital processes. According to Kolker and Story [12], typical urgent hospital issues are related to the capacity of existing infrastructure to provide healthcare services, the necessary medical personnel to achieve the best operational and service performance objectives, optimized personnel schedules to provide safe and efficient care for patients; patient flows affecting wait or throughput times, efficient allocation and use of resources, and forecasting patient demand or transaction volumes for the short and long term. All these issues impact the quality, time, cost, safety, and even sustainability of providing services in healthcare institutions [13]. These issues can be found in developed or emerging countries and the public and private sectors, which pose significant challenges in healthcare management. For example, challenges in countries such as the USA, the UK, Sweden, and Brazil focus on cost reductions or efficiency improvement due to stiff competition in the healthcare market [1]. Still, in others, problems refer to waiting times, service capacity, and service quality due to insufficient medical staff, infrastructure, and resources. Numerous examples of these cases can be found in the literature on existing challenges in hospital services such as admissions and discharge, triage, phlebotomy, sterilization and launderette, ambulance, X-ray and MRI, and others (see, for instance, [53–55]).

In the last two decades, diverse efforts have been developed to support the growing challenges healthcare services face worldwide, calling for the discipline of industrial engineering and operations management, as the root causes reside in processes and operations. For example, approaches such as lean healthcare and healthcare management engineering, originating from the manufacturing industry, have been used to address quality, time, cost, and safety issues using tools such as discrete simulations, process mapping, 5Ss, seven wastes, visual aids, continuous flow, error-proof devices, quick setups, and pull-Kanban systems, among others [2, 4, 56–58]. Therefore, substantial progress has been made in theoretical and methodological terms to address these challenges and to provide training and certificates to support healthcare providers and professionals in these tools. However, there is little work on teaching and learning about these topics at the undergraduate level in universities [59].

Therefore, there is an opportunity to move in this direction to develop learning experiences for engineering students and, more specifically, biomedical engineering students to expand their professional roles in the healthcare field. As the learning experiences proposed in this work emphasize active and experiential learning, relevant learning situations should be identified in healthcare and hospital services provision from their processes and operations perspective. In this work, these processes and operations are mapped according to their level of situational complexity and patient care criticality, following the work of Flood and Jackson to map organizational situations [60, 61].

The complexity of relevant study situations refers to the multiple participants or stakeholders, types of activities, allocated resources, and relationships involved in a real healthcare setting or process. The notion of complexity presents a requisite management capacity of people for problem-solving to improve existing conditions, communication, and coordination of participants to carry out their actions. Situations with a low level of complexity usually require processes involving deterministic or predefined types of behavior. In contrast, situations with a high level of complexity typically refer to scenarios in which the resulting configurations of interactions create a myriad of causal relations with multidimensional effects over time.

On the other hand, the criticality of situations refers to patient care regarding the involvement and participation of medical personnel and the impact of activities on patient health conditions [62, 63]. Situations of high levels of criticality involve direct medical attention, providing healthcare treatments to patients, ethical issues, and data protection regulations. Medium levels of criticality refer to situations that indirectly affect or relate to patients’ health, healthcare treatments, ethical considerations, and data protection regulations. Finally, low criticality concerns cases without impacting the patient’s health, medical treatments, ethical considerations, and data protection regulations.

Table 1 shows some examples of activities and processes assigned to their levels of complexity and criticality. For example, billing is mapped as a high-complexity and low-criticality process since it regularly involves integrating information (i.e., materials and service charges) and decision-making from different hospital departments (e.g., operating theatres, clinical laboratories, imaging and radiology, and food service) but has a low impact on the patient’s medical condition. In contrast, outpatient consultation is mapped as low complexity and high criticality, as it involves minimal patient-staff interactions across different departments. However, it is a crucial process to provide adequate medical treatments.

**Table 1:** Mapping processes in healthcare settings based on their level of situational complexity and the criticality of patient healthcare. These processes represent examples of potential study situations in learning experiences

		Criticality		
		*Low*	*Medium*	*High*
**Complexity**	*Low*	General cleaning	Food services	Outpatient consultation
	*Medium*	Public relations	Ward cleaning & laundry services	Sterilization & Ambulatory procedures
	*High*	Billing	Medical imaging	Accidents & Emergencies

This framework provides a tool to categorize relevant study situations, which can be used to conceptualize plausible learning experiences but are restricted by educational, medical, operational, legal, and ethical considerations. Additionally, the framework can help identify relevant study situations for biomedical engineering education purposes that require validation to comply with healthcare criteria and applicable regulations within hospitals and healthcare providers. Therefore, it is paramount to consider this limitation when selecting any foreseeable study situation.

## Methods

**Fig. 2:**
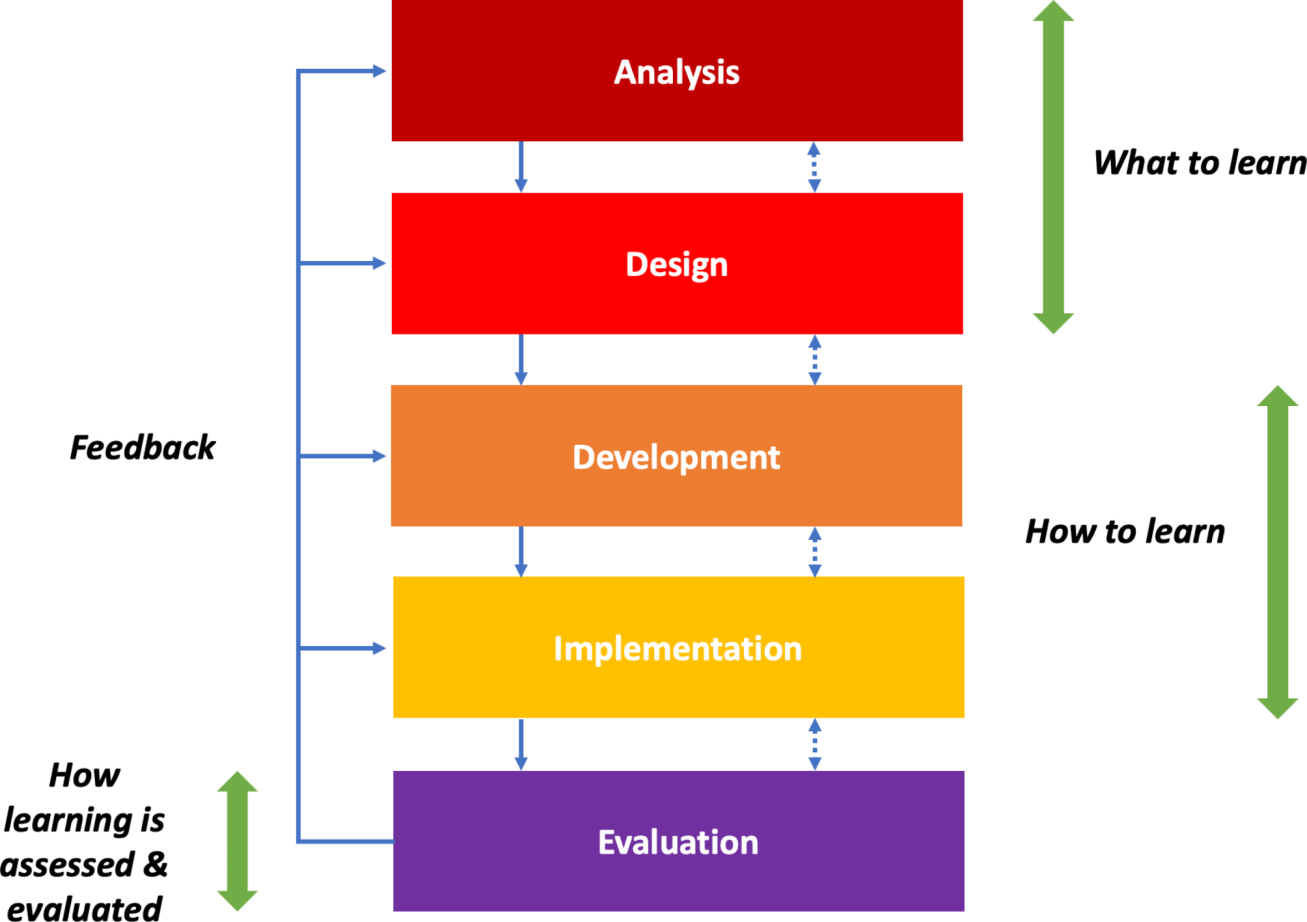
The ADDIE model for learning experiences. Adapted from [64]

This work aims to create relevant learning experiences for biomedical engineering education to develop transdisciplinary knowledge and skills to improve and optimize hospital and healthcare processes. Consequently, this work involves translating healthcare system processes into specific instructional designs based on experiential learning, answering the questions of *what to learn*, *how to learn*, and *how learning is assessed and evaluated*, in line with the constructive alignment proposed by Biggs and Tang [34]. In this case, the Analysis, Design, Development, Implementation, and Evaluation (ADDIE) model is proposed as a helpful method for the instructional design of learning experiences (Fig. 2) [64].

The ADDIE model provides high-level guidance for conceptualizing instructional designs following an iterative design process [65]. This model emphasizes the learner-centered approach as it focuses on supporting the active participation of students in their learning activities (e.g., problem-solving or decision-making). The model can be applied to various settings, given its systematic and generic structure. It also allows the identification of the needs of the target audience and reinforces the use of this information for the development, evaluation, and feedback of instructional designs to determine the effectiveness of the program. The ADDIE model provides an iterative process that considers corrective action or necessary changes and adaptations to deliver instructional designs successfully to meet the needs of the audience [66].

The *analysis stage* refers to the definition of the content of the course or module learning, the target group of learners, the learning objectives and outcomes, the format, the organization of the course or module, the teaching strategies, and the execution of the objectives. The *design stage* refers to the acquisition of knowledge by the students, the learning activities, and the measurement of the results of the learning outcomes. The *development stage* refers to instructional materials, learning infrastructure, and resources. The *implementation stage* points to teachers’ and students’ communications and interactions over the learning experience, either face-to-face, remote, online, blended, or hybrid, in their learning spaces. Finally, the *evaluation stage* consists of formative and summative assessments and evaluations of students and their learning achievements. It could also include the evaluation of the extent to which the expectations of the students were met in the learning experience on the relevance of knowledge, their interest and motivation, the development of skills and attitudes, the level of study, and others. All stages are interrelated, moving back and forth as required and feedback into each other, shaping the conceptualization and execution of a learning experience.

The following section presents the implementation of this method to exemplify its use and provide instances of learning experiences that show *what to learn*, *how to learn*, and *how learning is assessed and evaluated* for an expanded conceptualization of biomedical engineering education and also to give a reference for other engineering disciplines in higher education.

## Results

This section presents the results of implementing the ADDIE model as a method for the instructional design of experiential learning activities aimed at developing relevant knowledge and skills of biomedical engineering students to improve and optimize the hospital and healthcare process. Each subsection provides the results of applying each stage in the model.

### Analysis

The learning experiences described below were designed and implemented in an elective course on hospital management for last-year biomedical engineering undergraduate students at Tecnologico de Monterrey on the Campus of Mexico City. Ten students were enrolled in the course where these learning experiences were implemented.

Initially, this course aimed to introduce students to management in healthcare organizations. Consequently, the course covered general concepts of administration, leadership and team management, strategic planning, and quality management as applied to healthcare. As a learning outcome, students would develop managerial skills for human, technological, and strategic resources in health services.

However, the course was redesigned to introduce biomedical engineering students to concepts and tools for lean operations and management applied to healthcare. The new contents included value stream mapping (VSM), the seven wastes, root cause analysis, and the A3 methodology. The selection of new course content was based on a review of textbooks [2, 11, 56–58, 67] and training courses on lean healthcare and healthcare operations management, such as those offered by the Institute of Industrial and Systems Engineers and North Carolina State University and Johns Hopkins University in the USA, as well as on the professional experience of the authors in lean principles and tools. However, it was encouraging to see this selection reflected in a report on new opportunities for biomedical engineering education prepared by an international group of experts at WHO’s request [6].

Furthermore, while the original course used traditional teaching methods, the redesigned course purposefully incorporates experiential learning through problem-based learning (PBL).

### Design

The learning experiences described here were designed to involve students in analyzing and redesigning health operations for improvement and optimization. These experiences were designed to engage students in observing a real-life healthcare process, identifying the current and desired situation, defining the appropriate countermeasures to reach the latter, and preparing a deployment plan. These activities were carried out through *living application cases* (i.e., study cases in which students address an ongoing situation from a healthcare organization), described later in this subsection.

The PBL activities related to these learning experiences were organized into four project stages, each linked to a stage of the Kolb Experiential Learning Cycle (Table 2). These project stages are as follows:

1. Exploration (concrete experience). First, students familiarize themselves with the organization, particularly the department or service where the learning experience unfolds. This familiarization begins with a search for relevant information on the Internet (e.g., the organization’s official website). It becomes fully experiential when students visit the place and interview the involved staff members. More specifically, students familiarize themselves with the organization’s mission, vision, and values, the services offered by the specific department, its stakeholders (e.g., staff members and patients), and the key processes these services rely upon. Later, students conduct fieldwork to observe a healthcare process, shadow relevant stakeholders and document their observations.
2. Definition of the problem (reflective observation). Students collaboratively identify problems or issues in the current situation, its root causes, and the desired situation. They use some healthcare management engineering concepts and tools, such as value stream mapping, root cause analysis, and the A3 methodology.
3. Definition of an improvement plan (abstract conceptualization). Students define countermeasures to reach the desired situation and relevant performance metrics.
4. Definition of the deployment plan (active experimentation). Students prepare a deployment plan to guide the adoption of the proposed countermeasures. Implementing this plan lies beyond the student’s control, as it depends on the staff in charge of the process. However, students are encouraged to keep in touch with them to follow up on potential implementations and their impact.

**Table 2:** Experiential learning activities are organized in four project stages to cover the entire Kolb Experiential Learning Cycle

Learning cycle stage	Project stage	Description
Concrete experience	Exploration	Students observe healthcare processes involved in the problem, shadow relevant stakeholders, and document their findings.
Reflective observation	Definition of the problem	Students identify problems or issues in the current situation, its root causes, and the desired situation.
Abstract conceptualization	Definition of improvement plan	Students define countermeasures to reach the desired situation and relevant performance metrics.
Active experimentation	Definition of the deployment plan	Students prepare a deployment plan to guide the adoption of the proposed countermeasures.

Four application cases were identified through interviews with management, operational, and healthcare personnel from healthcare services and organizations (i.e., the training partners). These included the National Institute of Cardiology, the National Institute of Rehabilitation, and the Medical Service at the Tecnologico de Monterrey, the campus of Mexico City. The application cases identified were:

1. Improving the maintenance services offered by the Department of Biomedical Engineering of the National Institute of Rehabilitation.
2. Improving the services offered by the Sterilization and Disinfection Unit of the National Institute of Rehabilitation.
3. Improving the services offered by the Clinical Laboratory at the National Institute of Cardiology.
4. Improving the services offered to students and staff by the Medical Service at Tecnologico de Monterrey.

The work carried out by the students led to the identification of more specific projects, such as improving the corrective maintenance process offered by the Department of Biomedical Engineering at the National Institute of Rehabilitation by reducing waste or the blood-taking process provided to outpatients by reducing delays (Table 3).

**Table 3:** Application cases, specific student project examples, and the complexity and criticality of the process addressed

Application case	Student project example	Complexity	Criticality
Improving the maintenance services offered by the Department of Biomedical Engineering of the National Institute of Rehabilitation	Improving the corrective maintenance process	High	Medium
Improving the services offered by the Sterilization and Disinfection Unit of the National Institute of Rehabilitation	Improving the sterilization process offered to the orthopedic surgery service	Medium	Medium
Improving the services offered by the Clinical Laboratory at the National Institute of Cardiology	Improving the blood-taking process offered to outpatients	High	Medium
Improving the services offered to students and staff by the Medical Service at Tecnologico de Monterrey	Improving the emergency response process	High	High

### Development

#### Learning spaces

Learning spaces are traditionally considered physical places such as classrooms, workshops, laboratories, and libraries. However, the experiential learning approach described here requires more diverse venues or locations where learning can occur [42, 43]. Consequently, the experiential learning activities above included fieldwork in different services and departments of the National Institute of Cardiology and the National Institute of Rehabilitation in Mexico City. These institutes are the country’s reference for medical care, training, and research in their respective fields. Additionally, the Medical Service of the Tecnologico de Monterrey, the Campus of Mexico City, was also a venue where some students performed fieldwork.

#### Learning resources

##### *Teaching team*

The experiential learning experiences described above were jointly designed and developed by the Department of Biomedical Engineering and the Department of Industrial and Systems Engineering. Two faculty members (one from each department) were involved. Later, they also delivered the hospital management course. Therefore, this course involved transdisciplinary teaching and learning. Importantly, this initiative received strong support from the director of the biomedical engineering undergraduate program, whose perspective was critical to identifying its relevance to the professional prospects of biomedical engineering students.

##### *Time*

The hospital management course runs for 16 weeks with three hours per week for in-class activities (e.g., lectures, talks from guest speakers, and guided learning activities). In addition, students are expected to dedicate five hours per week to assignments outside of the classroom, including PBL-related activities. The teaching team also allotted five additional hours for activities outside the classroom (e.g., class preparation and student tutoring).

##### *Software*

A FlexSim Healthcare student license (FlexSim Software Products, Inc) was secured to allow students to simulate their process if required. FlexSim was initially developed for discrete-event simulation of manufacturing systems and processes using highly realistic 3D layouts. Later, it was adapted to FlexSim Healthcare to analyze and optimize patient-based processes. This software allows:

1. Creating a 3D layout to replicate the system’s look while maintaining exact spatial relationships so that travel and transport times are accurate.
2. Building the model, i.e., declaring the workflow and its properties (e.g., process times).
3. Analyzing the model through several different graphs and charts.
4. Optimizing the process by testing different ‘what if’ scenarios.

A search in Google Scholar using the query (“flexsim healthcare” AND “education” AND “Mexico”) did not produce results regarding the use of FlexSim software to create learning experiences in biomedical engineering education in Mexico. Therefore, this work contributes to advancing biomedical engineering education in the country.

### Implementation

Students and faculty members had face-to-face interactions at least three hours per week during the term (sixteen weeks). These interactions were mainly through lectures and guided learning activities in the classroom. Further interactions were held during tutoring, with each faculty member dedicating up to five hours per week to this activity.

Furthermore, students also had face-to-face interactions during guided learning activities and assignments, including PBL-related activities.

Finally, the students interacted with the training partners and related stakeholders during fieldwork (i.e., interviews and shadowing).

### Evaluation

The evaluation of the learning process is twofold. First, it involves formative and summative assessments of the student’s learning achievements. Second, it refers to the evaluation students make of their learning experience. These evaluations complement each other to provide a broader perspective on students’ learning results and perceptions of the course. However, more evaluations are required to conclude the impact of this type of learning experience.

#### Assessment of experiential learning activities

A formative assessment of all students enrolled in the course was conducted after each of the three first project stages (i.e., exploration, project definition, and improvement plan definition). These assessments were based on a cumulative written report, updated with the information produced in each stage. These assessments allowed the teaching team to give students timely and relevant feedback. Furthermore, a summative assessment was performed after the fourth project stage (i.e., deployment plan definition). This summative assessment was based on two deliverables. Firstly, a presentation where students communicate their findings and proposals to the faculty and the person in charge of the department or service. Secondly, a final report that captures all the elements of the four stages.

#### Evaluation of the students’ learning experience

Data on the student learning experience and other critical aspects related to the course were collected at the end of the course. These data were collected using the Student Opinion Survey (SOS), an anonymous and voluntary internal feedback survey with fourteen closed-ended and one open-ended question. Closed-ended items use a 5-point rating scale, with 5 representing the highest value (e.g., the highest level of agreement). The open-ended question asks students to estimate the average number of hours they devoted in addition to class hours per week. Table 4 shows an extract of the SOS focused on the questions most relevant to the evaluation of the learning experiences of the students. Although this survey inquires about the course as a whole, the number of hours devoted to experiential learning activities makes it reasonable to attribute a strong influence of the latter on student responses. In addition, the survey might not be considered a sampling tool but an instrument to provide information on the students’ views about their course.

**Table 4:** Extract of the Student’s Opinion Survey, an anonymous internal survey, applied at the end of the course. The included questions are considered more relevant to evaluating students’ learning experience

Item key	Item description
RTP	The professor implemented learning activities that allowed students to link the course content with real-life situations.
SAP	The professor provided students with follow-up and tutoring in their individual and collaborative learning process.
INN	The professor innovated the learning experience by including activities and resources that added value to it.
HDS	How many hours per week did you devote to this course beyond classroom activities?

Unfortunately, only one in ten students voluntarily responded to the SOS (i.e., a response rate of 10%). This student rated very highly (i.e., “totally agree”) on the questions RTP, SAP, and INN and declared devoting eight hours per week in addition to class hours to complete course assignments (including PBL-related assignments). Needless to say, no conclusions, inferences, or generalizations can be drawn from these results. They are only included here to illustrate how the course evaluation is done.

## Discussion

The proposed instructional design of transdisciplinary learning experiences benefited students, faculty, and training partners. Students engaged with their local and broader community through fieldwork at the Medical Service of Tecnologico de Monterrey and three different departments at two of Mexico’s National Institutes of Health. These learning experiences allowed students to observe a real-life healthcare process (concrete experience), identify the current and desired situation (reflective observation), define the appropriate countermeasures to reach the latter (abstract conceptualization), and prepare a deployment plan (active experimentation). Therefore, the students went through the entire Kolb experiential learning cycle, addressing the question of *how to learn*. On the other hand, students engaged in health operations analysis and redesign for improvement and optimization through four application cases, addressing the question of *what to learn*. Finally, students underwent formative and summative assessments and were encouraged to complete a course evaluation survey addressing *how learning is assessed and evaluated*. These learning experiences allowed biomedical engineering students to develop transdisciplinary knowledge and skills and a broader professional role within healthcare institutions to become facilitators of process improvement, affecting value delivery. This professional role aligns with the biomedical engineering role recently proposed by the WHO [6]. In this case, biomedical engineering professionals should expand their professional role in healthcare institutions beyond medical equipment and infrastructure to involve process improvement and optimization and the construction of response capacity in healthcare systems. Anecdotally, one of the students engaged in these learning experiences has played several leadership roles related to improving healthcare processes during her career as a clinical engineer. Although this case does not prove a positive impact of the learning experiences above on her professional profile, it supports the idea that biomedical engineering professionals are called to play those roles.

From the faculty perspective, these learning experiences resulted in an improved teaching experience due to the intellectual challenge it represents in mentoring students under transdisciplinary experiential learning approaches and the motivation derived from it. Indeed, the application cases addressed during the course were equally new to students and faculty. Therefore, guiding students through the project stages was more challenging than mentoring them under traditional learning approaches. In general, faculty members gained experience working on real-life problems with students, explored new disciplinary applications for the biomedical engineering profession, challenged their knowledge by crossing their discipline boundaries, and established meaningful conversations with training partners. The instructional design and learning experiences also led faculty members to take learning activities outside the classroom and the university to redefine their understanding of learning spaces and environments in real-world scenarios and settings that contextualized disciplinary knowledge beyond technical engineering aspects.

From the perspective of the training partner, healthcare providers gained a deeper understanding of the dynamics of their services and processes without investing considerable time and resources. Anecdotally, one of the training partners adopted some of the students’ proposals to test their capacity to reduce wait times in one of its healthcare processes (namely, outpatients’ blood taking).

However, this teaching-learning experience presented challenges for students and faculty alike. By design, this course requires students to devote eight hours of study per week. According to the only student who responded to the SOS, they dedicated eight hours per week in addition to class hours to complete the assignments (including PBL-related assignments). Although that was the answer of a single student, faculty members assert that number based on their observations over the course. From the faculty perspective, designing and implementing these learning experiences was also time-consuming. They had to start preparing for the experiences around five months in advance and devoted five hours a week to tutoring the students during the course. This represents a significant effort that, in some higher education institutions, could be justified only if it were to benefit a substantial number of students. However, scaling up this teaching and learning approach would create the additional challenge of securing more case studies, which could be manageable with students working in groups.

The students also faced challenges during fieldwork. For example, some students had to arrive early (6 a.m.) to shadow and interview outpatients queuing outside the hospital. Some other students had to deal with healthcare personnel who were reluctant to share information with them and skeptical about the potential benefits of doing so. These challenges were verbally reported during tutoring sessions, progress reviews, and final presentations.

This work also has some limitations. First, the amount of data collected to assess the impact of the proposed learning experiences was limited due to the low response rate of the end-of-course survey to students. This prevents us from quantitatively assessing this teaching-learning experience, which does not devalue the reporting and qualitatively evaluating the latter. However, more work can be done to quantitatively assess the impact of these learning experiences on variables such as student interest, motivation, perceived relevance, and learning outcomes.

A related and more fundamental limitation of this work arises from the uniqueness of learning experiences and their report as case studies; that is, neither the students, the learning scenario, nor any other element of the experience will be the same from one implementation to another. This uniqueness makes it difficult for researchers to compare results from different implementations. However, further instances of instructional designs could be developed to identify patterns that allow the elaboration of more stable statements and conclusions about the use of experiential learning and the ADDIE model for instructional design and their impact on student learning outcomes for biomedical engineering education.

Finally, we identify a few directions for this work to evolve. First, the proposed learning experiences must be adapted to new curricular developments (e.g., updates to the biomedical engineering curriculum) using state-of-the-art instructional methods, such as service and challenge-based learning, or problem-solving methodologies, such as design thinking. In addition, new learning experiences can be created in the wider variety of learning spaces supported by state-of-the-art technologies, fostering a ubiquitous learning [22]. Furthermore, the assessment of students’ learning could be improved by incorporating a tool for students to track and reflect on their learning process, such as a regular learning journal, to foster mandatory reflection. Finally, this type of work allows higher education to contribute to the education of “integration experts,” defined as experts who lead, administer, manage, monitor, assess, accompany, and advise others on integration within inter and transdisciplinary projects [68].

## Conclusions

This work contributes to advancing biomedical engineering education through experiential learning in healthcare engineering by proposing the design and implementation of transdisciplinary and situated learning experiences.

The transdisciplinary nature of these learning experiences expands the conceptualization of biomedical engineering education based on the contemporary challenges faced in healthcare to enrich the role of biomedical engineering professionals in their practice. Thus, it contributes to the increased readiness of biomedical engineering professionals for the demands of healthcare systems of the 21st century [6, 14].

Furthermore, the contextualized nature of these learning experiences is supported by fieldwork conducted in learning spaces beyond the traditional classroom, highlighting the need to rethink what makes or defines learning spaces [42].

Some transdisciplinary and situated learning experiences were exemplified in this article using existing frameworks for learning processes and instructional design (Kolb’s Experiential Learning Cycle and the ADDIE model, respectively [35, 36, 64]). How we use them will hopefully guide other educators when designing and implementing their learning experiences. Further implementations of these learning experiences are required to conclude their impact on students’ learning and career prospects.

## Data Availability

All data generated or analyzed during this study are included in this published article.
